# The Effect of Ca and Mg Concentrations and Quantity and Their Correlation with Caries Intensity in School-Age Children

**DOI:** 10.1155/2018/2759040

**Published:** 2018-05-08

**Authors:** Milaim Sejdini, Kastriot Meqa, Nora Berisha, Ekrem Çitaku, Nora Aliu, Sokol Krasniqi, Sami Salihu

**Affiliations:** ^1^Department of Orthodontics, Faculty of Medicine, University of Prishtina, Prishtina, Kosovo; ^2^Department of Periodontology and Oral Medicine, Faculty of Medicine, University of Prishtina, Prishtina, Kosovo; ^3^Department of Orthodontics, University Dentistry Clinical Center of Kosovo, Prishtina, Kosovo; ^4^Department of Maxillofacial Surgery, Faculty of Medicine, University of Prishtina, Prishtina, Kosovo

## Abstract

**Introduction:**

Saliva is a watery product formed by the salivary glands and secreted in the mouth. Besides the fundamental factors, saliva with its ingredient is one of the main etiologic factors of caries presence. In the development of dental caries, the relationship between demineralization and remineralization is influenced by the presence of saliva, which facilitates the transportation of ions, oral bacteria, and fermentable carbohydrates to the exposed surfaces of teeth. The main components of saliva electrolytes are sodium, calcium, copper, magnesium, bicarbonates, and organic phosphates. Increase in calcium level in the remineralization solution may enhance the deposition velocity of minerals in the caries lesion. Magnesium, except the similar role as calcium, takes an active part in cellular reparation process.

**Materials and Methods:**

In this study were included students of age 12-13. Students were divided into three groups; the first group included caries-free children, the second group with DMF = 1–6, and the third group with DMF > 6. Fully stimulated and nonstimulated saliva was collected in sterile graded patches in the morning hours, due to the circadian rhythm in 5-minute length. Chemical analyzes have been conducted at the Faculty of Chemistry, Ss. Cyril and Methodius University in Skopje. Statistical processing has been performed at the Medical Faculty, at the Institute of Medical Statistics in Skopje.

**Results:**

Depending on the DMF, before and after stimulation, regarding Ca level, no significant differences were found. However, the Ca level prior to stimulation is significantly higher in the second group compared to the third one. Also, the amount of Ca after stimulation in the first group was significantly higher. No significant differences in Mg level and amount were found prior to and after stimulation, while the amount of Mg after stimulation had a significant difference between groups.

**Conclusion:**

Saliva mineral analysis has shown significant differences in quantitative and qualitative components between examining groups. The decrease of calcium molality in saliva might play a significant role in caries occurrence; thus, we may suppose that saliva calcium level significantly influences hard dental tissues defense mechanism. Magnesium levels after stimulation showed a significant difference between groups I and III and no significant differences between groups I and II. Magnesium level and amount correlate with calcium level, favoring elemental caries resistance.

## 1. Introduction

Saliva is a watery substance formed in the mouth, secreted by the salivary glands. Human saliva contains 99.5% water, but also many important substances, including electrolytes, mucus, antibacterial compounds, and various enzymes. The joint action of factors in derived full saliva provides a multifunctional defense system, which might fail only if the salivary flow is greatly reduced [1].

Saliva contributes to the digestion of food and to the maintenance of oral hygiene. Without normal salivary function, the frequency of dental caries, gum disease, and other oral problems increases significantly. We still do not have reliable diagnostic procedures to predict such a particular risk in the pediatric and adolescent age. Although many questions remain unanswered, however, it is commonly acknowledged that saliva and the influence of its components are important for oral health.

There are many different agents within saliva and plaque that serve to protect the tooth surface against caries development. Salivary flow rate, buffering capacity, antimicrobial activity, microorganism aggregation and clearance from the oral cavity, immune surveillance, and calcium phosphate binding proteins, all interact to inhibit or reverse demineralization of exposed tooth surfaces.

Saliva contains a supersaturated solution of calcium and phosphate, which neutralizes acids. Many factors might affect the quantity and quality of saliva. Secretion of saliva is under the control of the autonomous nervous system, which controls both the volume and type of saliva secreted. Mixed and unstimulated saliva derives from a general secretive system of oral cavity glands in the unstimulated environment, where no dietary substance influences taste or other receptors in the oral cavity. It is thought that caries varies on the following two inseparable variables: enamel and its surrounding environment. Most of the electrolytes are found in all types of salivary glands, although their level may differ within various glands. Secretion of saliva may be stimulated or triggered mechanically, dietarily, electrically, or pharmacologically; furthermore, it relies on cholinergic and beta-adrenergic glandular stimulation. There is an obvious difference in salivary electrolyte levels, deriving from different sources. The parotid saliva contains more phosphate and less Ca^+2^ ions compared to mixed saliva. On the contrary, the level of Ca^+2^, Mg^+2^, and Zn^+3^ in mixed saliva is significantly higher, compared to the parotid one.

In terms of the relationship between dietary mineral intake and oral disease, the calcium (Ca) and phosphorus (P) concentrations of dental plaque and the levels of Ca and P ions in the saliva could affect the balance between demineralization and remineralization of enamel [2].

Some epidemiological studies have revealed that humans with relatively high Ca and P in their plaque experience correspondingly lower caries [3]. Higher Ca concentration of plaque is associated with low caries incidence [4].

Dental caries is a microbial disease of the calcified tissues of the teeth, characterized by demineralization of the inorganic portion and destruction of the organic substance of the tooth. There is no universally accepted opinion of the etiology of dental caries.

Demineralization and remineralization have a crucial impact on the hardness and strength of tooth enamel. The battle to keep teeth strong and healthy is dependent upon the ratio between demineralization and remineralization. Demineralization occurs at a low pH when the oral environment is undersaturated with mineral ions, relative to a tooth's mineral content. The enamel crystal, which consists of carbonated apatite, is dissolved by organic acids (lactic and acetic) that are produced by the cellular action of plaque bacteria in the presence of dietary carbohydrates. Loss of Ca ions (phosphorus and fluoride) of supersaturated saliva enables demineralizing tissue recovery. Increasing Ca concentration in the remineralizing solution can increase the rate of deposition of minerals in the lesion. Increasing Ca level within a remineralizing solvent may increase mineral incorporating-layering velocity. In the development of dental caries, the relationship between demineralization and remineralization is influenced by the presence of saliva, which facilitates the transportation of ions, oral bacteria, and fermentable carbohydrates to the exposed surfaces of teeth. It is this complex interrelationship that we must understand better in order to fight the battle of maintaining tooth integrity.

Magnesium (Mg) may also play an important role in preventing periodontal disease and caries as it has the unique ability to reduce inflammation caused by bacterial toxins [5].

Similarly, Mg has also been shown to have both significant [6] and no significant associations with tooth decay [7].

## 2. Aim

The aim of this research is to determine Ca and Mg amount and concentration in unstimulated and stimulated saliva with children at high risk of caries with DMF > 6 and DMF = 1–6 and with the group of caries-free children, to determine if there is a correlation between the Ca and Mg amount and concentration compared to the caries frequency between the three investigated groups, to find the effect of Ca and Mg values in the saliva in preventing caries, and to determine the correlation between concentration and amount of Ca and Mg in unstimulated and stimulated saliva in the three investigated groups of children.

## 3. Materials and Methods

Student examination was performed in school settings, involving children of 12-13 years of age. The survey comprised 1248 students. Subjects were divided into three groups; first group listing caries-free ones (DMF=O), the second with DMF = 1–6, and the third one listing those with DMF > 6. Out of the total number of subjects (106), the first group comprised 25 subjects, the second group 47, and the third one 34. For examinee classification purposes, in the first group, the WHO method on intensity index of measured caries was utilized. Furthermore, oral hygiene maintenance awareness with children groups at this age is satisfactory. With all examinees, full-blended and nonstimulated saliva was studied. All examinees' saliva samples were collected in graded sterile tubes, in the morning hours from 8:00 to 9:00, before intake, at least an hour after brushing. Saliva was collected for 5 minutes, soaked with pipe injector out of mouth flour, and spilled into a sterile tube. Total saliva in strict time frames was collected. During examination time, the patients were calm, sitting, and not allowed to swallow. After a while, sterile paraffin to chew was given, for the same exact 5-minute period. Collection of stimulated saliva and other related procedures were conducted in an identical manner as with the nonstimulated one. All tubes collected (212) were cultured at −20°C, enabling bacteria and enzyme inactivation, thereof probably influencing the biochemical and immunochemical analysis.

Chemical and immunochemical tests have been conducted in the Faculty of Science, Ss. Cyril and Methodius University in Skopje. Analyses were performed by flame atomic absorption spectrometer model Solaar S4 from Thermo Elemental (UK); for Ca analysis, we used the wavelength of 422.7 nm, spectral slit of 0.2 nm, and lamp current of 10 mA, while for Mg analysis, the wavelength of 285.2 nm, spectral slit of 1.0 nm, and lamp current of 4 mA, which represents a method with relatively high sensitivity. For the analysis of Ca and Mg, 2 ml of saliva samples were diluted with redistilled water in the volumetric flask of 25 ml by adding few drops of concentrated nitric acid.

Data were statistically processed at the Institute of Medical Statistics, Faculty of Medicine in Skopje. Average values, standard deviations, confidence interval, Mann–Whitney *U* test, Kruskal–Wallis H test, *t*-test, Wilcoxon matched pair (Z), analysis of variance (F), and so on were determined.

## 4. Results

**Figure 1:**
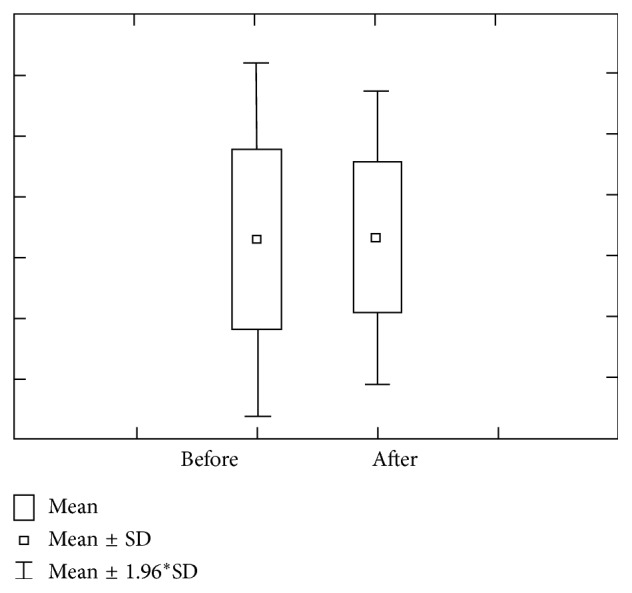
Concentration and quantity of Ca before stimulation for the first group of investigated subjects (DMF = 0).

**Figure 2:**
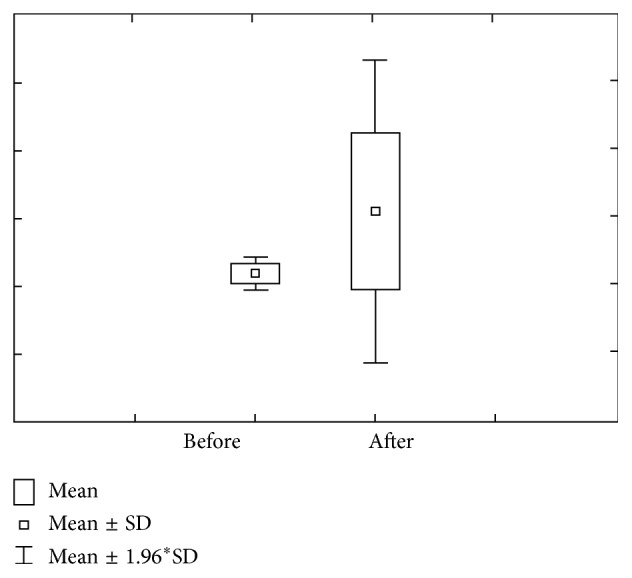
Concentration and quantity of Ca after stimulation for the first group of investigated subjects (DMF = 0).

Figures 1 and 2 show concentration and amount of Ca before and after paraffin stimulation. The concentration of Ca prior to stimulation in the first group varies within 1.29 ± 1.48 mmol/L ranges, with 0.67–1.90 confidence interval. The concentration of Ca after stimulation varies within 1.30 ± 1.22 mmol/L ranges, with 0.80–1.81 confidence interval. Ca amount prior to stimulation varies within 1.80 ± 1.23 *μ*mol/L ranges, with 1.29–2.31 confidence interval. Ca amount after stimulation varies within 10.91 ± 11.47 *μ*mol/L ranges, with 6.17–5.65 confidence interval.

**Figure 3:**
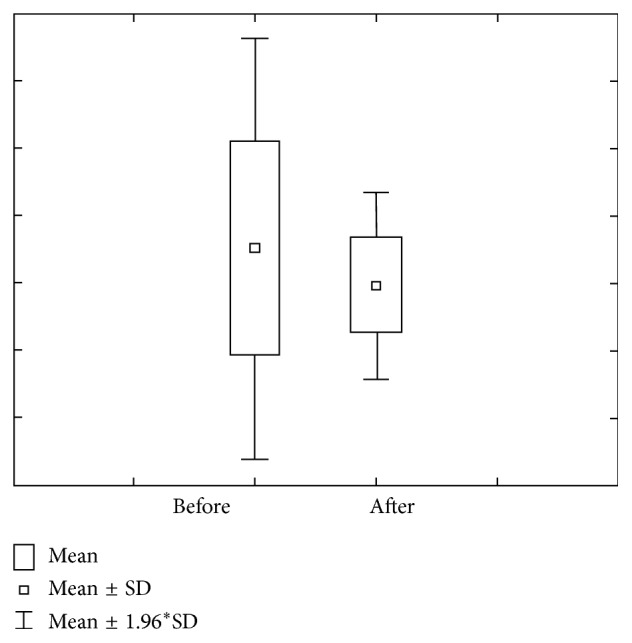
Concentration and quantity of Mg before stimulation for the first group of investigated subjects (DMF = 0).

**Figure 4:**
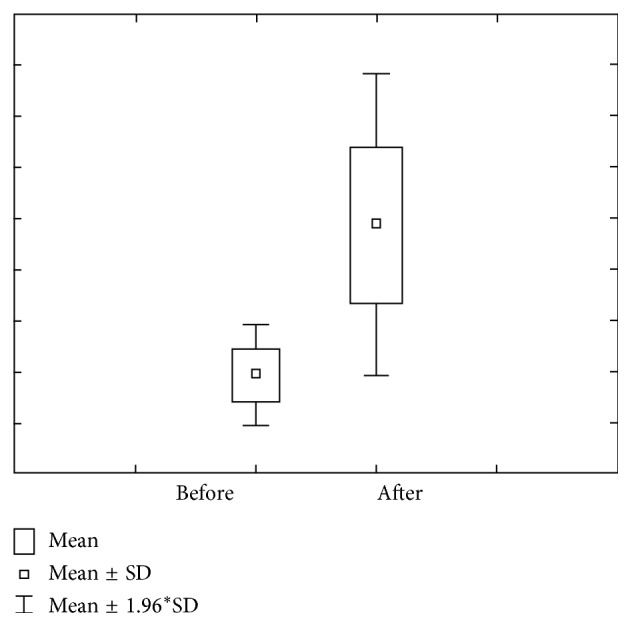
Concentration and quantity of Mg after stimulation for the first group of investigated subjects (DMF = 0).

Figures 3 and 4 show concentration and quantity of Mg before and after stimulation. We can notice that concentration of Mg before the stimulation level varies within 0.12 ± 0.07 mmol/L ranges, with a 0.09–0.15 confidence interval. Mg concentration after stimulation varies within 0.09 ± 0.03 mmol/L ranges, with 0.08–0.11 interval of confidence.

Mg amount prior stimulation varies within 0.18 *μ*mol/L ranges, with 0.14–0.23 interval of confidence. Mg amount after stimulation varies within 0.77 ± 0.30 *μ*mol/L ranges, with 0.65–0.90 interval of confidence.

**Figure 5:**
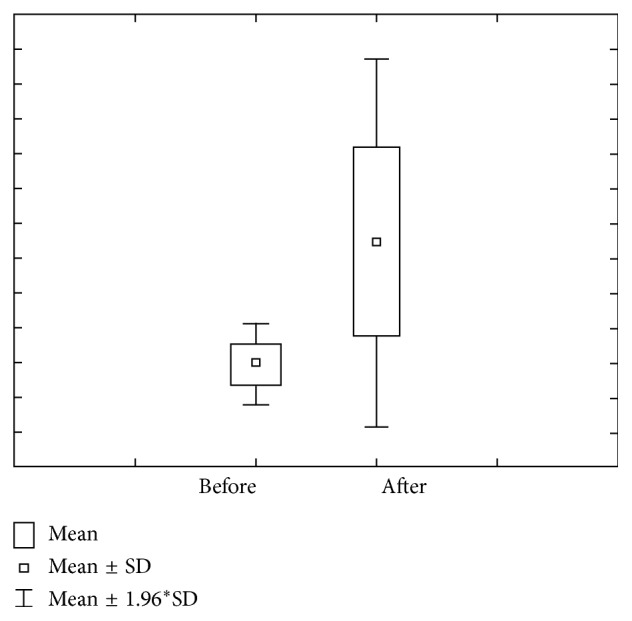
Concentration and quantity of Ca before stimulation for the second group of investigated subjects (DMF = 1–6).

**Figure 6:**
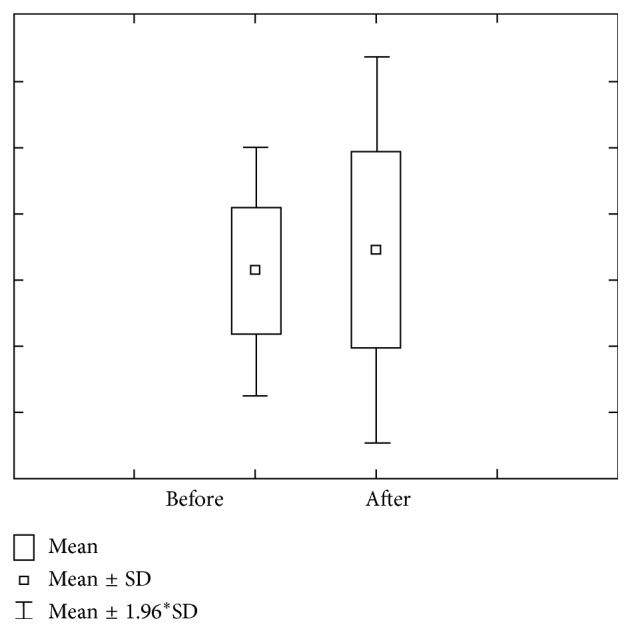
Concentration and quantity of Ca after stimulation for the second group of investigated subjects (DMF = 1–6).

The second group (DMF = 1–6) comprises 47 (44.34%) of total examinees. Figures 5 and 6 show the value of concentration and amount of Ca before and after stimulation. The concentration of Ca prior to stimulation varies within 1.07 ± 0.47 mmol/L ranges, with a 0.93–1.21 confidence interval. The concentration of Ca after stimulation varies within 1.23 ± 0.74 mmol/L ranges, with a 1.01–1.44 interval of confidence. Ca amount prior to stimulation varies within 1.95 ± 1.18 *μ*mol/L ranges, with a 1.60–2.29 interval of confidence, while Ca amount prior to stimulation varies within 8.95 ± 5.39 *μ*mol/L ranges, with a 7.37–10.54 interval of confidence.

**Figure 7:**
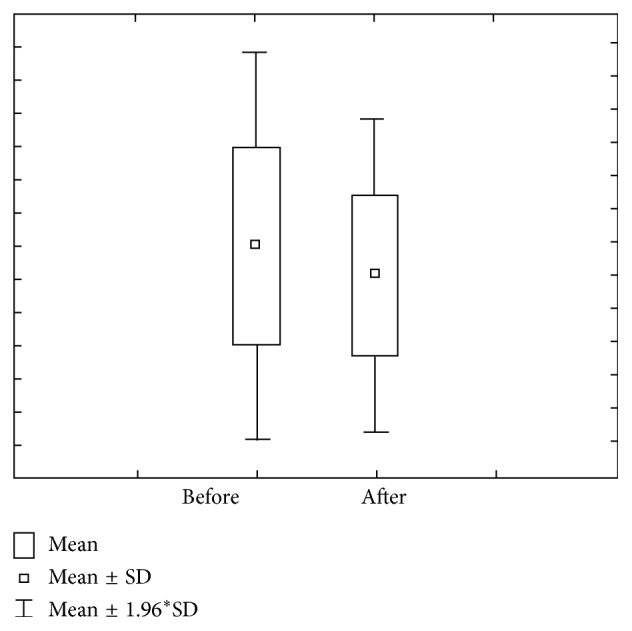
Concentration and quantity of Mg before stimulation for the second group of investigated subjects (DMF = 1–6).

**Figure 8:**
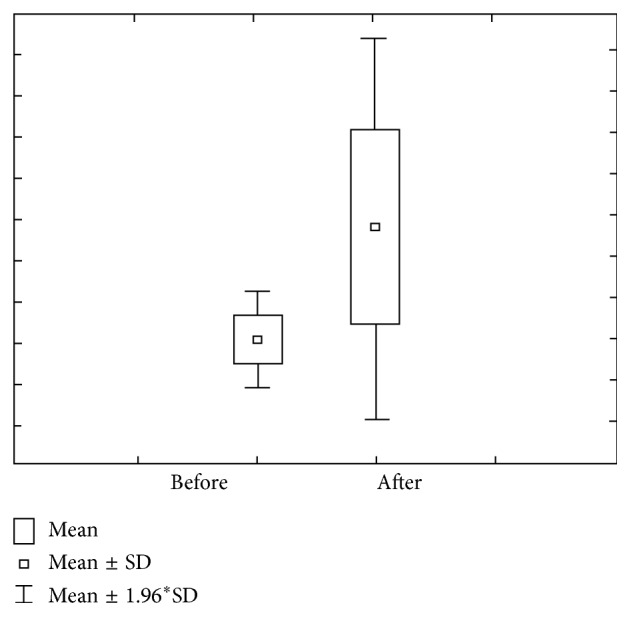
Concentration and quantity of Mg after stimulation for the second group of investigated subjects (DMF = 1–6).

Figures 7 and 8 show the value of concentration and amount of Mg before and after stimulation. Concentration of Mg prior to stimulation varies within 0.11 ± 0.05 mmol/L ranges, with 0.10–0.13 interval of confidence. The concentration of Mg after stimulation varies within 0.10 ± 0.04 mmol/L ranges, with 0.08–0.11 interval of confidence. Mg amount prior to stimulation varies within 0.21 ± 0.12 *μ*mol/L ranges, with 0.17–0.24 interval of confidence, while Mg amount prior stimulation varies within 0.75 ± 0.47 *μ*mol/L ranges, with 0.61–0.88 interval of confidence.

**Figure 9:**
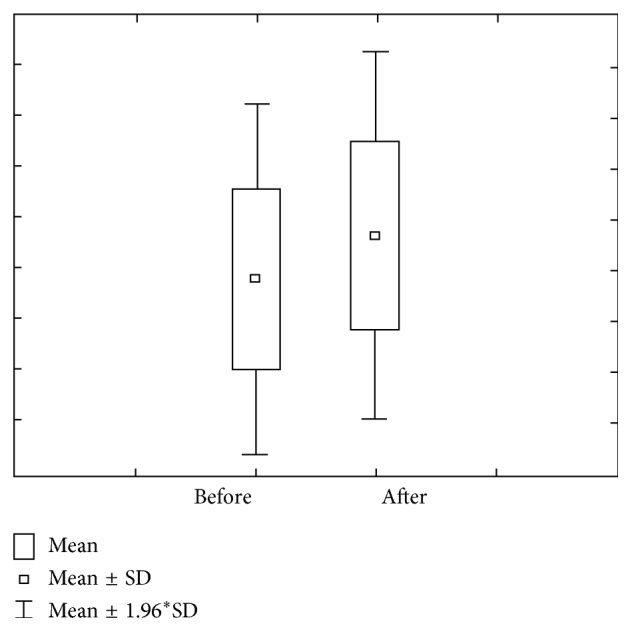
Concentration and quantity of Ca before stimulation for the third group of investigated subjects (DMF > 6).

**Figure 10:**
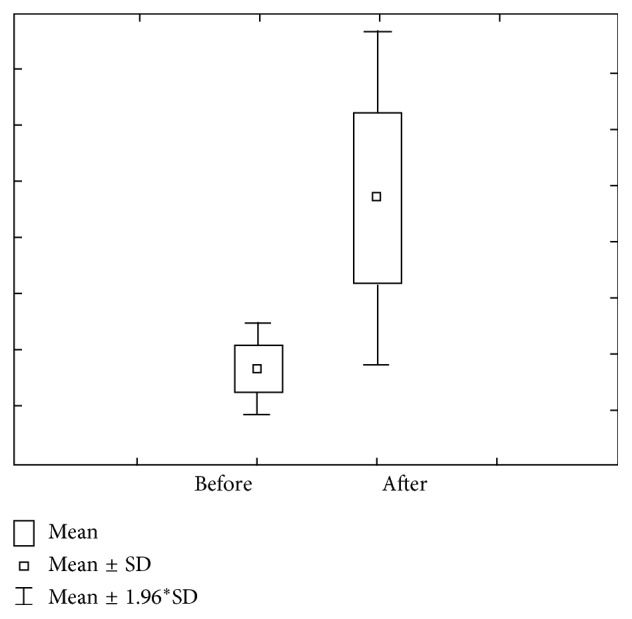
Concentration and quantity of Ca after stimulation for the third group of investigated subjects (DMF > 6).

Figures 9 and 10 show the value of concentration of Ca before and after stimulation in group 3 (DMF > 6). Concentration of Ca prior to stimulation varies within 0.95 ± 0.35 mmol/L ranges, with 0.83–1.08 interval of confidence. The concentration of Ca after stimulation varies within 1.13 ± 0.36 mmol/L ranges, with 1.00–1.26 interval of confidence. Ca amount prior to stimulation varies within 1.38 ± 0.82 *μ*mol/L ranges, with 1.09–1.66 interval of confidence, while Ca amount after stimulation varies within 7.50 ± 3.01 *μ*mol/L ranges, with 6.44–8.55 interval of confidence.

**Figure 11:**
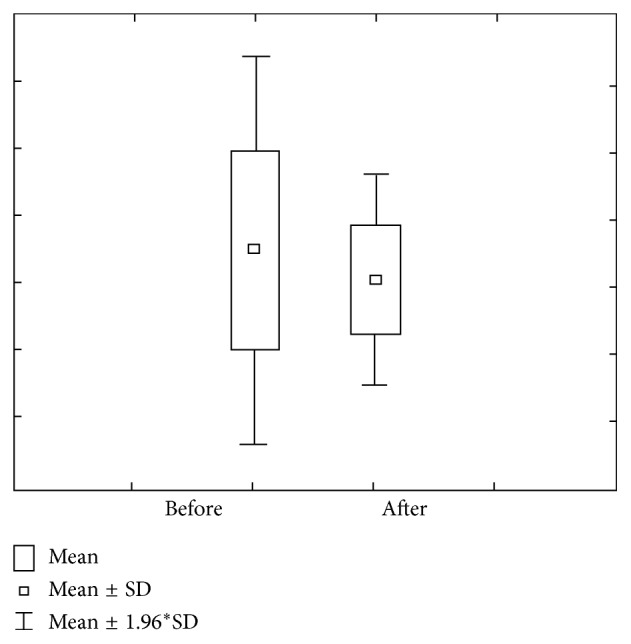
Concentration and quantity of Mg before stimulation for the third group of investigated subjects (DMF > 6).

**Figure 12:**
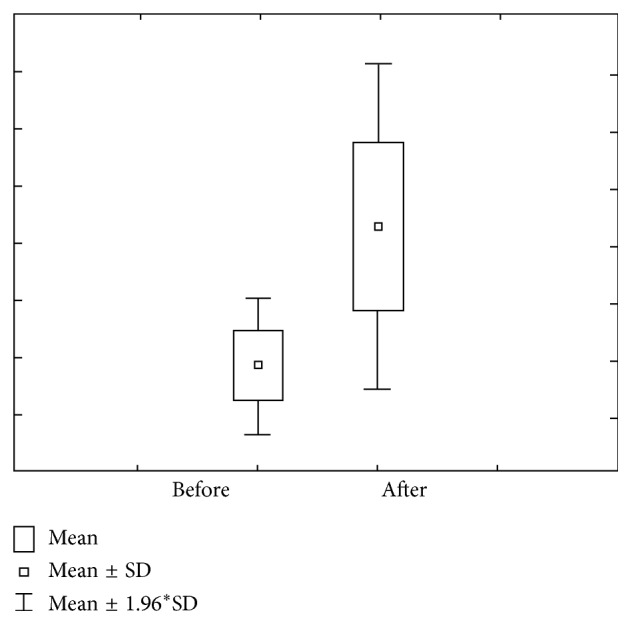
Concentration and quantity of Mg after stimulation for the third group of investigated subjects (DMF > 6).

Figures 11 and 12 show the concentration of Mg before and after stimulation for the third group of the research. Concentration of Mg prior to stimulation varies within 0.12 ± 0.07 mmol/L ranges, with 0.09–0.15 confidence interval. Concentration of Mg after stimulation varies within 0.10 ± 0.04 mmol/L ranges, with 0.08–0.11 confidence interval. Mg amount prior to stimulation varies within 0.17 ± 0.12 *μ*mol/L ranges, with 0.13–0.21 confidence intervals, while Mg amount prior to stimulation varies within 0.56 ± 0.76 *μ*mol/L confidence intervals.

**Figure 13:**
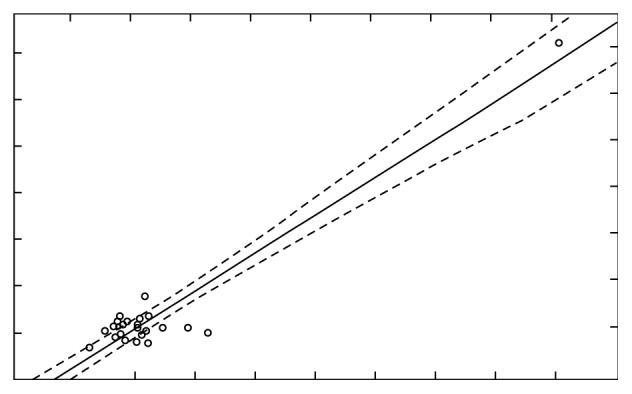
Concentration of Ca before and after stimulation with correlation *r*=0.94889.

**Figure 14:**
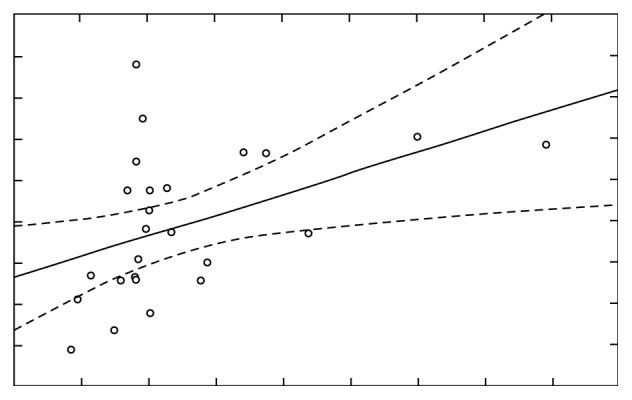
Concentration of Mg before and after stimulation with correlation *r*=0.45492.

The comparison of Ca concentration before and after stimulation, for *r*=0.94 (*p* < 0.05), indicates the presence of very high correlation (Figure 13), while the comparison of Mg concentration before and after stimulation, for *r*=0.45 (*p* < 0.05), indicates the presence of moderately strong correlation (Figure 14).

**Figure 15:**
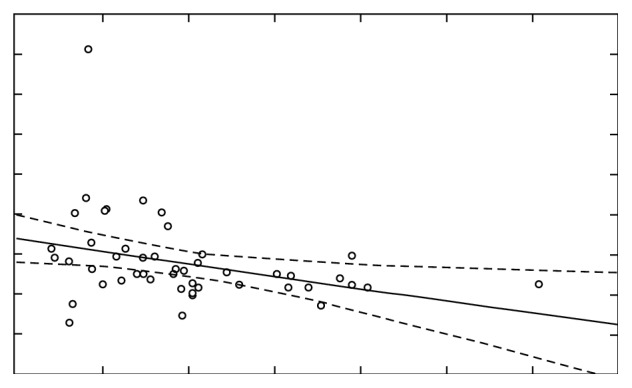
Quantity of Ca before and after stimulation, correlation *r*=−0.3332.

**Figure 16:**
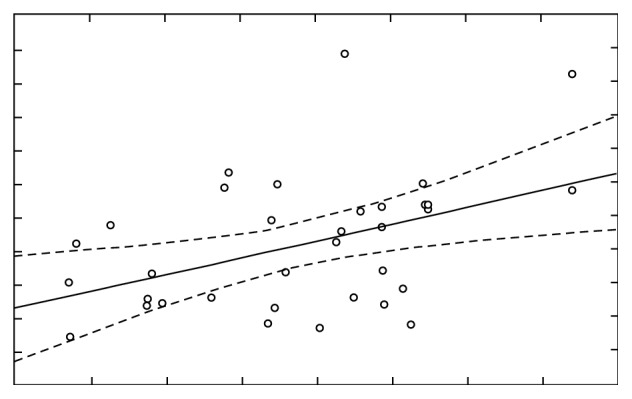
Concentration of Ca and Mg before and after stimulation, correlation *r*=0.43042.

A comparison of Ca before and after stimulation for *r*=−0.33 (*p* < 0.05) shows the presence of a weak negative correlation (Figure 15), while the researched report between the concentration of Ca concentration before stimulation and concentration of Mg after the stimulation for *r*=0.43 (*p* < 0.05) indicates the presence of moderately high correlation (Figure 16).

## 5. Discussion

There are quantitative differences in the relative proportions of human saliva electrolytes in the major salivary gland secretions. For instance, parotid saliva is relatively low in calcium and high in phosphate as compared to submandibular and sublingual secretions.

Research outcome of native saliva mineral composition has shown major physiological differences in the composition of some minerals in all study groups [8–10] correlating to our results. Caries development, among other things, is in a firm relation to physical and chemical properties of dental enamel, as well as to physical, biochemical, and immunochemical properties of the tooth surroundings. Using electromicroscopic analysis, it is found that established remineralizing solution, consisting of Ca, P, and F in an extracted tooth, submerged in artificial saliva and afterward in the abovementioned solution, recovers enamel damage (caries, decay) and enhances the remineralizing potential of saliva [11]. It is generally accepted that saliva secretion and its components are of major significance for dental and overall mouth health [12].

Calcium role as fluoride-calcium [13] composition shows a great stability within the oral environment, thanks to the superficial absorbance of HPO_2_ in a crystal surface, as well as to the establishment of melting phase, limited phase, which can be used as a reservoir with controlled pH of enamel and dental plaque ions [14].

In a study of the Ca and P composition in full and stimulated saliva, it is found that patients with low caries frequency have higher amounts of Ca ions compared to those with higher caries prevalence, which matches our results too. The saliva of examined students at the age of 12, affected with severe caries, was characterized with alteration of Ca homeostasis, companioned with Ca frequency redistribution within nonstimulated saliva [15]. In this case, mild deviation of the overall Ca level was noticed. Saliva level of Ca plays a significant role in the dental hard tissue defense system. On the contrary, lower Ca levels in the saliva have enhancing force for hydroxylapatite precipitation in neutral pH and have the larger force to dissolve hydroxylapatite in even more lower critical pH [16].

Calcium-enriched saliva enables initial damage (caries) remineralization. As mouth rinsing with Ca-enriched saliva fights caries, stimulation of saliva secretion via chewing gum is proved to be efficient too.

Undersaturation deriving from acidic bacteria production results in caries, while other way round, supersaturation can help in remineralization of white spots [17]. In a study of electrolyte levels, among students with low caries activity and those with caries inclusiveness from 6 to 14, the critical quantitative difference was found [9].

Our study resulting from statistical tests show that, in the saliva of students with caries, there is a significant correlation between Ca and Mg levels. It is thought that this was important for proper mineralization, but also, it is foreseen that for maturation purposes, higher levels are needed [18]. Studies carried out on mineral composition of nonstimulated native saliva are few, moreover, with contradictory results. These oscillations of electrolyte physiological volume sphere are induced by optimal secretion velocity and by glandular secretion composition alteration, with the following key players: gender, day period, diet, emotional, and health condition.

A higher value of the deft index was associated with lower amounts of daily Ca or P intake. Because the associations were no longer significant after adjusting for potential confounding factors, one could speculate that the daily dietary intakes of Ca or P might not be the primary nutritional factors for caries [19].

The decrease in Ca levels results in a reduction of enamel crystallinity, increasing one's retentive surface and decreasing overall resistance. In a study of saliva Ca levels, carried out on 23 caries-affected students and 32 unaffected ones, it is found that Ca levels in noncariotic students are far higher, compared to cariotic ones [10]. In a caries intensity-related study of qualitative composition and secretion of mixed saliva in a group of 264 students (6–14 y.o.), the correlation was found between saliva Ca composition, qualitative secretion, and teeth resistance against caries. Calcium levels in serum and saliva are decreased in caries-prone individuals. Our findings are a complete match to this study.

Regarding studies carried out on Ca molality of mixed saliva in caries-affected and not affected students, the data obtained from our study are matching Liappis and Konstantine findings [9] and not matching Tokyeva [20] and Show et al. [10] findings. A study conducted on 70 healthy patients, in collected saliva samples, Mg composition was analyzed in three different study groups: 7–14 y.o., 18–28 y.o., and 48–60 y.o. Significant age-related Mg level difference is found between groups I and II, as well as between groups I and III [21]. The composition and the level of inorganic ions in dental plaque and saliva are significantly influencing the initiation and development of caries via saturation level alteration in the watery phase surrounding dental enamel. The level of inorganic ions of Ca and Mg differs significantly between plaque liquid and saliva [22]. This means saliva and plaque liquid differ in inorganic composition, probably due to plaque bacteria metabolic activity. Regarding correlation coefficient between dental plaque and saliva composition, a significant correlation between Mg ions and not Ca ions are found. As previous studies claim, Mg continuously incorporates in dental tissues, while simultaneously, the same amount is lost through attrition, and thus, no deficiency occurs. Since Mg through ionic exchange can substitute Ca, loss of crystal density may occur, explaining the increase of caries predisposition in an environment with a higher level of Mg than Ca.

Reduction of crystal density inside enamel is in direct correlation to higher level Mg and CO_2_ ions [23]. In a prospective study of saliva Mg, with the spectrophotometric atomic absorption of 186 patients treated with digoxin, it is found that salivary Mg level is correlated with the digoxin level in plasma; an obvious increase of salivary Mg level was induced due to digoxin therapy [21]. The amount of salivary Ca and Mg is correlated to alteration of the same mineral levels in blood serum; thus, Ca infusion administration did not change the overall Ca and other mineral levels in saliva [14].

## 6. Conclusion

In this study, we can conclude that

- the Ca concentration before and after saliva stimulation and the quantity of Ca before and after saliva stimulation have shown a high correlation. Calcium level significantly influences hard dental tissues defense mechanism;
- calcium level after stimulation with *Z*=0.76, *p* > 0.05, is altered significantly compared to the same ones prior to stimulation. With the increase of caries number, the calcium level decreases;
- the level and the composition of Mg have been monitored; it is calcium-related and goes in favor of elemental caries resistance;
- the relationship between the concentration of Mg before and after saliva stimulation and the relationship of Mg quantity before and after saliva stimulation have shown a medium-high correlation;
- the Mg level, even after stimulation, did not show significant alterations. The level of Mg for *t*=9.90 and *p* < 0.001 was significantly higher after stimulation, while Mg level between groups after stimulation was increased, and a significant difference was found between groups I and III and not between groups I and II;
- the amount of Ca before and after saliva stimulation and the amount of Mg before and after saliva stimulation have shown a low negative correlation;
- the level of Ca and Mg in caries sensitive children could have been appropriate in order to maintain the balance between demineralization and remineralization of the enamel;
- sensitivity of the tooth to caries may be due to differences in chemical composition of the enamel structure.
